# An improved single-step lysis protocol to measure luciferase bioluminescence in *Plasmodium falciparum*

**DOI:** 10.1186/1475-2875-11-42

**Published:** 2012-02-10

**Authors:** Sandra Hasenkamp, Eleanor H Wong, Paul Horrocks

**Affiliations:** 1 Institute for Science and Technology in Medicine, Keele University, Staffordshire ST5 5BG, UK; 2 Infection and Immunity and Wellcome Centre for Molecular Parasitology, Glasgow Biomedical Research Centre, 120 University Place, Glasgow G12 8QQ, UK

**Keywords:** Luciferase, Single-step lysis, Stage-specific, Promoter function, *pcna*

## Abstract

This report describes the optimization and evaluation of a simple single-step lysis protocol to measure luciferase bioluminescence from genetically modified *Plasmodium falciparum*. This protocol utilizes a modified commercial buffer to improve speed of assay and consistency in the bioluminescence signal measured by reducing the manipulation steps required to release the cytoplasmic fraction. The utility of this improved assay protocol is demonstrated in typical assays that explore absolute and temporal gene expression activity.

## Background

The use of reporter assay systems in the human malarial parasite *Plasmodium falciparum *have proven an invaluable tool in the functional characterization of *cis*-acting sequences that govern the promoter and terminator activities of gene flanking sequences [1-3]. The two most commonly used reporter systems utilize either a bacterial chloramphenicol acetyltransferase (*cat*) gene or a *Photinus pyralis *luciferase (*luc*) gene [4,5]. Both assay systems offer attractive properties in terms of sensitivity and large linear range for quantification of activity. The non-isotopic bioluminescence assay provided by *luc*, however, offers additional attributes such as speed of assay (no need for lengthy incubations for the end-point of the *cat *assay), easier handling and waste disposal characteristics, and ability to exploit recent advances in instrumentation for the detection of bioluminescence. One final advantage of the *luc *assay, its flexibility, has recently been evidenced in the malarial field by the development of a high throughput anti-malarial drug screening assay using transgenic parasites expressing *luc *[6-9].

There are, however, inherent limitations in the utilization of the *luc *reporter assay system. Whilst valuable in demonstrating the effect of modified gene flanking sequences in determining the absolute level of gene expression, the concatamerization of episomal plasmids, unequal segregation of plasmids during mitotic division, and frequent use of mis-matched 5' and 3' gene flanking sequences, limit their use in the exploration of molecular mechanisms that govern temporal patterns of gene expression [1,10]. These limitations, however, can be mitigated by the use of matched 5' and 3' flanking sequences in combination with the *bxb1 *integrase system to ensure production of homogenous populations of parasites that each contain a single reporter gene cassette [11,12]. However, the repeated lysis and washing steps required to produce a cleared parasite cytoplasmic fraction from the intraerythrocytic stages of parasite development often generate a variation in the bioluminescence signal that needs to be carefully controlled (typically through cell count and protein concentration quantification). This variation is particularly confounding when attempting a multi-variant analysis, resulting in data that may demonstrate a trend but falls short of demonstrating significance.

Here the optimization and validation of a modified luciferase reporter gene assay protocol for the asexual intraerythrocytic stages of *P. falciparum *is described. This revised protocol uses a simple single-step lysis protocol with an improved lysis buffer formulation to generate a more consistent bioluminescent signal output. The utility of this modified assay is demonstrated by showing its ease of use in generating a comprehensive portrait of temporal gene expression and how consistency in data can provide significant information from a multi-variant promoter deletion study where previously only trends had been described.

## Methods

### *Plasmodium falciparum *clones and culture

The genetically modified *P. falciparum *clone expressing luciferase (Pfluc) used here has previously been described [12]. This clone was originally derived from the AHE1 clone (Dd2^attB^) using the mycobacteriophage *bxb*1 integration system [11,12]. In Pfluc, the *luc *reporter gene is flanked by 1417 bp and 647 bp of 5' and 3' flanking sequences, respectively of *Pfpcna *(PF13_0328). Clones Δ2 and Δ3 contain 1160 bp and 990 bp of 5' *Pfpcna *flanking sequence, respectively [12]. All clones were cultured under standard conditions (complete growth medium, 2% haematocrit, atmosphere of 1% O_2_, 3% CO_2_, and 96% N_2_) and maintained using 5 nM WR99210 and 2.5 μg/ml BSD (blasticidin S) drug selection [11]. Staging and parasitaemia was determined by Giemsa-stained thin blood smears and light microscopy. Synchronization of cultures was attained using sequential sorbitol treatment [13].

### Standard reporter lysis assay

*Plasmodium falciparum*-infected erythrocytes (IE) were collected by centrifugation (5 min, 3000xg, RT) and 20 μl samples of packed IE lysed using 10 volumes of 1× phosphate buffered saline (PBS)/0.1% saponin at room temperature for 5 min. Following centrifugation, the parasite pellet was washed three times in 1× PBS and re-suspended in 100 μl of 1× Reporter Lysis Buffer (Promega), and subjected to three cycles of freeze (liquid nitrogen) and thaw (37°C) with a final centrifugation step of 13000 rpm for 2 min to clear the parasite supernatant. Bioluminescence readings were measured using the single-tube luminometer (Glomax 20/20, Promega) from 20 μl of cleared parasite supernatant and 100 μl of Luciferase Substrate Reagent (Promega).

### Statistics

Analysis for significance in the bioluminescent signals determined for the reporter deletion series was carried out using n = 6 independent measurements for each construct at each developmental stage and analysed using one-way analysis of variants (ANOVA), with Tukey's post tests for significance, on MiniTab version 14. All other experiments were carried out using n = 5 independent measurements. Where indicated, t-tests were carried out in GraphPad Prism version 5.0 (GraphPad Software, San Diego California USA).

## Results

### Evaluation of parameters for a simplified single-lysis step luciferase reporter assay

The standard luciferase reporter assay protocol for *P. falciparum *requires release of the parasite from the host erythrocyte, extensive washing of the parasite pellet to remove haemoglobin, and then a repeated process of freeze-thaw lysis of the parasite to release the cytoplasmic fraction [5,12]. This protocol was devised prior to the improved performance and sensitivity of modern luminometers and it was reasoned that a revaluation of the lysis conditions should be explored in an attempt to simplify the protocol to use only a single lysis step.

**Figure 1:**
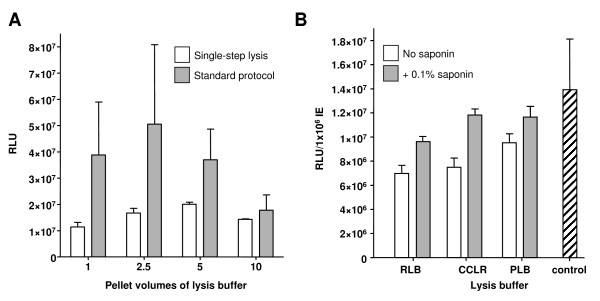
**Comparison of performance of the standard and single-step lysis protocols**. (**A**) The mean and stdev (n = 5) of the relative luminescent units (RLU) from 20 μl of packed IE subject to the indicated pellet-volumes of 1 × RLB is shown. The key indicates the protocol used for each assay. (**B**) Comparison of bioluminescent signal derived using single-step lysis protocol for the indicated buffer ± 0.1% saponin supplement. As a control (hatched bar), the same IE culture was subject to 1 × RLB and the standard lysis protocol.

Throughout this study, a standard of five repeats of 20 μl of packed trophozoite-IE were prepared to investigate each set of parameters under investigation. Following lysis, 20 μl of IE or isolated-parasite supernatant fractions were mixed with 100 μl of luciferase assay substrate (Promega, UK) and the bioluminescent signal measured for 10 sec using a Glomax 20/20 luminometer (Promega). The signals obtained from the standard lysis protocol were initially compared with those from a single-step lysis of IE using a commercially available reporter lysis buffer (RLB, Promega, UK). Here, packed IE were re-suspended in a range of pellet volumes of 1 × RLB (1, 2.5, 5 and 10-fold pellet volumes), mixed using a bench-top vortex and incubated at room temperature for 5 min. A 20 μl volume of IE supernatant was then removed for detection of the bioluminescent signal. This single-step lysis protocol is thus dramatically less complex and time-consuming than the previous standard protocol, which typically takes 30-45 min. Whilst the standard lysis protocol, irrespective of the volumes of RLB used, always generated a higher bioluminescent signal, the variation in signal obtained for the four matched samples was high; such that no significant difference could be determined between each of the conditions used (Figure 1A). This is in marked contrast with the improvement in consistency of bioluminescent signal derived using the simpler single-step lysis protocol; where a significant difference in all pair-wise comparison was determined (*t*-test, *p *< 0.05).

To explore the possibility of improving the bioluminescent signal obtained using the single-step lysis protocol, two variables were explored. First was the use of different lysis buffers, and second the pellet-volume of lysis buffer used. A range of commercial lysis buffers optimized for bioluminescence assays are available; here the performance of the previously used RLB was compared against cell culture lysis reagent (CCLR, Promega, UK) and passive lysis buffer (PLB, Promega, UK), in each case assessing its use in the presence or absence of a 0.1% saponin supplement. Using five-pellet volumes (i.e. 100 μl) of each lysis buffer, the bioluminescent activity of the same IE culture was assayed and compared to a control using the standard lysis protocol and a five-pellet volume of RLB (Figure 1B). Saponin was observed to significantly enhance the bioluminescent signal obtained using all lysis buffers (*t*-test, p < 0.005). Comparison of the bioluminescent signal using the different buffers shows a clear preference for the modified PLB/0.1% saponin buffer. A revised assay was thus adopted using this modified buffer as the data would indicate that some 65% of the bioluminescent signal lost on adoption of the single-step assay can be recovered without adversely compromising the consistency of the data produced.

**Figure 2:**
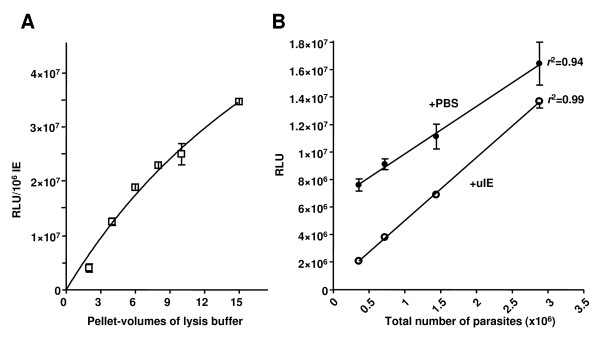
**Optimization of the single-lysis step protocol**. (**A**) The mean ± stdev of RLU normalized for parasite number is compared to the pellet-volumes of PLB/0.1% saponin lysis buffer. (**B**) Comparison of the mean ± stdev RLU against total parasite number shows a strong linear correlation. Bioluminescence was measured from varying volumes of IE pellet supplemented with either PBS (filled circles) or uIE (unfilled circles) to a total volume of 20 μl.

To explore the effect of the pellet-volume of lysis buffer used, the same trophozoite stage Pfluc IE culture was subjected to single-step lysis using between 2-15 fold pellet volumes of PLB/0.1% saponin. As 20 μl of IE supernatant is used to measure bioluminescence, this signal output was normalized for an equivalent number of parasites to account for the dilution attributed to the use of different volumes of lysis buffer across the experiment. This normalization reveals a slight hyperbolic curve (near linear, r^2 ^= 0.90) relationship between the bioluminescent signal per standardised parasite number and the volume of lysis buffer, suggesting that larger buffer volumes are more efficient in releasing luciferase from the IE pellet (Figure 2A). Saturation of the bioluminescent signal was only consistently achieved using in excess of 20-pellet volumes of PLB/0.1% saponin (data not shown). Given this near linear relationship over the fold-volumes explored here and consideration of the cost/benefit of complete lysis and dilution of bioluminescent signal when using large volumes of this commercial reagent; five volumes of PLB/0.1% saponin would appear to balance these competing variables and was taken forward for all subsequent studies described here.

The final step in the validation of the revised single-step lysis protocol was a demonstration that the bioluminescent signal generated is directly proportional to the number of parasites being assayed. A range of different volumes of Pfluc IE (2-16 μl) were subject to single-step lysis using PLB/0.1% saponin and the bioluminescent signal plotted against parasite number (Figure 2B). To explore the effect of haemoglobin from uninfected and infected erythrocytes on this relationship, the volume of the IE pellet was made up to 20 μl using either phosphate-buffered saline (PBS) or packed uninfected erythrocytes (uIE). The bioluminescent signal obtained from IE supplemented with uIE was, in all cases, significantly less than that when supplemented with PBS. Plotting the bioluminescence signal against a linear scale of the number of parasites, reveals a strong correlation between IE number and signal for both conditions; with trend lines that would appear to be converging as the total number of erythrocytes (uIE and IE) becomes equivalent. This presumably reflects the known quenching effect of haemoglobin on luciferase bioluminescence [14]. Whatever assay condition applied, however, this revised single-step lysis protocol produces a bioluminescent signal directly proportional to the number of parasites irrespective of the presence of haemoglobin and would indicate that washing away of haemoglobin is not required.

### Demonstrating the utility of a single-step lysis protocol to investigate absolute and temporal changes in gene expression

**Figure 3:**
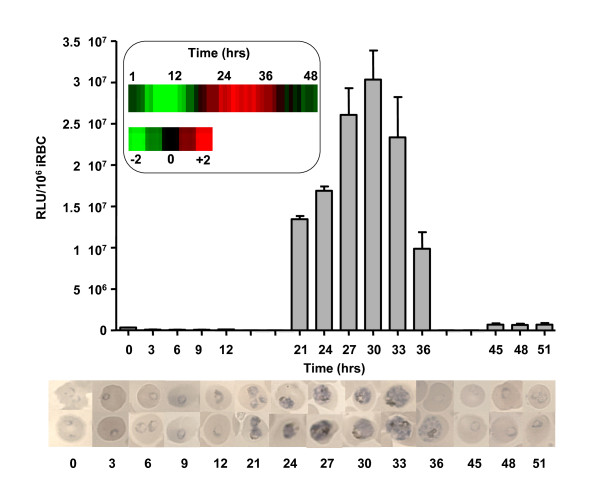
**Temporal regulation of the *Pfpcna *promoter during intraerythrocytic development**. Starting from an early ring stage culture (*c*. 3-6 hpi), samples were removed periodically over a 51-hour period and the bioluminescent signal normalized for parasite number measured. Images from Giemsa-stained thin smears of the time points investigated are shown to demonstrate the major morphological stages present. The inset box shows a 48-hour "phasogram" microarray image from the Malaria IDC comparison database for *Pfpcna *as well as a key to represent log-fold up (red) and down (green) accumulation of the steady state transcript level.

The *luc *reporter gene in Pfluc is flanked by 1417 bp and 647 bp of 5' and 3' flanking sequences, respectively, of the *Pfpcna *(PF13_0328) gene [12]. *Pfpcna *encodes the proliferating cell nuclear antigen, a protein essential for efficient DNA polymerase δ activity during S-phase. These flanking sequences have previously been demonstrated to be sufficient to completely reconstitute the trophozoite-specific temporal pattern of intraerythrocytic expression of *Pfpcna *over the *luc *reporter gene [12,15,16]. Previous analysis of the temporal pattern of luciferase expression in Pfluc, however, was focussed on a small number of samples harvested only from major morphological stages of intraeythrocytic development. The ease of use of the single-step lysis protocol was exploited here to facilitate a more comprehensive analysis of the temporal profile of luciferase expression across a full cycle of IE development (Figure 3). Over a 51 h period, samples were harvested at 14 time points from a starting culture of tightly synchronized ring-stage Pfluc. As expected, the onset of the *Pfpcna*-mediated bioluminescent signal was linked to the development of early trophozoite stage parasites and peaked in mature trophozoites; entirely coincident with the expected demand for *Pfpcna *during schizogony [12,15]. The expression levels start to subside as morphological evidence of schizogony is evident, with the bioluminescent signal essentially absent in the re-invaded rings. Comparison of the temporal luciferase reporter gene activity with the temporal pattern of *Pfpcna *steady-state mRNA levels (Figure 3 inset), available from microarray assays [17], show the previously described correlation between steady-state mRNA levels and luciferase expression, providing comprehensive evidence for the complete recapitulation of temporal expression using the *Pfpcna *flanking sequences described.

**Figure 4:**
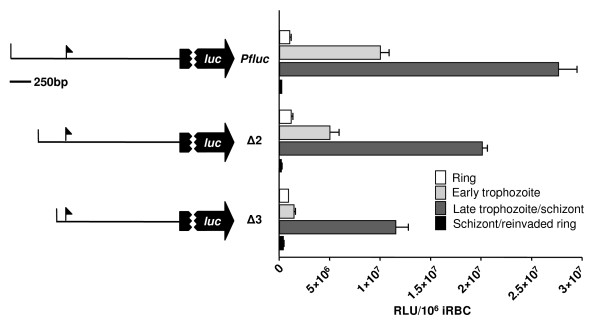
**Stage-specific analysis of *Pfpcna *promoter deletions**. The mean and stdev (n = 6) RLU normalized for parasite number from four time points during intraerythrocytic development (see key and main text) are plotted adjacent to a schematic that illustrates the promoter construct investigated. An arrow indicates the mapped transcription start site and details of sizes of flanking sequences are in methods.

Functional characterization of the *Pfpcna *promoter has identified two distal regions that affect promoter activity, both of which were shown to competitively bind *trans*-acting nuclear factors [12,16]. These studies, whilst able to derive significant differences between the bioluminescent signals produced from the full-length and minimal promoter constructs, were, however, unable to demonstrate significance for an intermediate promoter construct due to variance in the bioluminesce signals obtained using the standard promoter assay employed in that study. Moreover, the preferred analysis of this type of promoter deletion study would include samples isolated at different morphological stages to explore both temporal and absolute changes in promoter function. This multi-variate statistical analysis, however, requires a ready method to assay multiple samples and consistent datasets. In an attempt to show whether the single-step lysis protocol could establish significance in such a study, all three parasite clones (Pfluc, Δ2 and Δ3 [12]) used in the previous study were tightly synchronized to ring stage parasites and the bioluminescent signals monitored at ring (six-14 hours post-infection, hpi), early trophozoite (18-24 hpi), late trophozoites/schizont (30-36 hpi) and schizont/reinvaded rings (42-two hpi) stages. As expected, the peak of bioluminescence in all clones occurs in the mature trophozoite/schizont stage as these promoter deletions do not appear to affect the temporal activity of the *Pfpcna *promoter, only its absolute activity (Figure 4). And, as expected, as additional functional components of the promoter region are deleted, there is a contemporaneous decrease in luciferase activity. However, using the improved consistency in bioluminescent signals measured here, significant differences could be established between the absolute activity of the different promoter constructs in both the early trophozoite and late trophozoite/schizont time points (*p *< 0.001). Whilst comparisons in the other samples were not significant, this presumably results from what appears to be the very low, probably background, bioluminescent signals at these time points.

## Conclusion

Here a revised luciferase reporter gene assay protocol is described, based on the use of a single lysis-step, which offers a simpler and quicker procedure whilst delivering a more consistent bioluminescent signal. Reduction in the bioluminescent signal due to the presence of haemoglobin does not affect the specificity of the assay, and this loss in signal can be ameliorated using a saponin-supplementation of a commercial buffer. The utility of this revised assay in typical studies that examine the absolute and temporal properties of *P. falciparum *promoters is demonstrated. It is anticipated, however, that this protocol could be more widely applied to bioluminescence reporter gene assays in other intraerythroctytic pathogens.

## Competing interests

The authors declare that they have no competing interests.

## Authors' contributions

SH and PH designed the study which was predominantly carried out by SH. EHW provided initial material and performed the statistical analyses. All authors read and approved the final manuscript.
